# OLDER ADULT VOLUNTEER AND PEER EDUCATOR EXPERIENCES COLLABORATING ON INTERVENTION RESEARCH

**DOI:** 10.1093/geroni/igad104.1344

**Published:** 2023-12-21

**Authors:** Enid Rubio, Odell Allen

**Affiliations:** University of South Florida, Tampa, Florida, United States; University of South Florida, Tampa, Florida, United States

## Abstract

The final two speakers will present on their experiences, benefits, and challenges of collaborating on both trials described previously as an older adult volunteer coach or peer educator. One speaker is a DMFB Coach trained and certified in the DMFB intervention for depressed older adults, who delivered the intervention to five English-speaking older adults and is in the process of delivering the intervention to five Spanish-speaking older adults. One speaker is a Peer Educator for the hospital trial, trained and certified in a peer educator intervention for older adults after hospitalization, to support their transition and self-management at home. Each speaker will discuss their experiences delivering their respective interventions, including an overview of the training, certification, and supervision processes, as well as benefits and challenges. Benefits include the satisfaction of helping others, connecting with other coaches and peer educators, and learning technical skills and the intervention. Challenges have included technical issues related to virtual delivery, which are being overcome through technical assistance from the study teams; balancing the expectations of the program with other personal responsibilities, which are managed through discussion with supervisors and the study team being as flexible as possible, such as with scheduling; and occasional challenges with clients, which are addressed through regular and ad hoc supervision. The speakers will end with recommendations for other study teams that plan to work with older adults delivering behavioral interventions.

